# Association of sexual dysfunction according to *DSM-5* diagnostic criteria with avoidance of and discomfort during sex in a population-based sample

**DOI:** 10.1093/sexmed/qfad037

**Published:** 2023-07-14

**Authors:** Thula U Koops, Verena Klein, Ramona Bei der Kellen, Jürgen Hoyer, Bernd Löwe, Peer Briken

**Affiliations:** Institute for Sex Research, Sexual Medicine, and Forensic Psychiatry, University Medical Centre Hamburg-Eppendorf, Hamburg 20246, Germany; Institute for Sex Research, Sexual Medicine, and Forensic Psychiatry, University Medical Centre Hamburg-Eppendorf, Hamburg 20246, Germany; Department of Psychology, University of Southampton, Southampton, United Kingdom; Epidemiological Study Center, University Medical Centre Hamburg-Eppendorf, Hamburg 20246, Germany; Institute for Clinical Psychology and Psychotherapy, Dresden University of Technology, Dresden 01062, Germany; Department of Psychosomatic Medicine and Psychotherapy, University Medical Centre Hamburg-Eppendorf, Hamburg 20246, Germany; Institute for Sex Research, Sexual Medicine, and Forensic Psychiatry, University Medical Centre Hamburg-Eppendorf, Hamburg 20246, Germany

**Keywords:** DSM-5, population-based study, prevalence, sexual dysfunction

## Abstract

**Background:**

Sexual dysfunction frequencies and diagnostic indicators among older populations are relevant for public health measures, but evidence from large population-based studies is still scarce.

**Aim:**

To determine sexual dysfunction frequencies for women and men according to the *Diagnostic and Statistical Manual of Mental Disorders* (fifth edition; *DSM-5*) among 45- to 74-year-old participants of the population-based Hamburg City Health Study and the factors associated with sexual dysfunction diagnoses.

**Methods:**

We determined sexual dysfunction frequencies according to the *DSM-5* and the factors associated with sexual dysfunction diagnoses (quality/avoidance of and discomfort during sex) among 45- to 74-year-olds from 7786 participants of the population-based Hamburg City Health Study. We analyzed cross-sectional self-report questionnaire data collected between 2016 and 2019 using descriptive statistics, comparative tests (Fisher test, Mann-Whitney *U* test), and logistic regression.

**Outcomes:**

Outcomes included sexual dysfunction frequencies - specifically, sexual difficulties experienced frequently or more often, over at least six months in the last year, causing severe or very severe distress, and not associated with physical health or relationship problems - and items on quality/avoidance of and discomfort during sex.

**Results:**

Participants’ median age was 62.0 years (IQR, 14) and 51.1% were women. The frequency of sexual dysfunction according to the *DSM-5* was 9.3% (95% CI, 8.3%-10.4%) in women and 6.2% (95% CI, 5.4%-7.1%) in men, with women’s sexual interest/arousal and men’s erectile disorder being most common. Sexual dysfunction rates increased with age: whereas complaints were more frequent among women than men in the younger groups, participants aged ≥65 years with a sexual dysfunction were more often men. Quality/avoidance of and discomfort during sex were significantly associated with a diagnosis of sexual dysfunction.

**Clinical Implications:**

Results suggest that sexual dysfunction caused by other than physical health or relationship factors is important to consider in this population. In addition, the avoidance of, perceived quality of, and discomfort during sex serve as valuable diagnostic indicators for the presence of sexual dysfunction.

**Strengths and Limitations:**

This study draws on robust data from a large sample to give valuable insight on the frequency of sexual difficulties and dysfunctions as defined by *DSM-5* criteria. Limitations represent the restriction to self-report questionnaire data, the focus on participants living in a metropolitan area, and the lack of experience of sexual difficulties due to a lack of sexual activity not being taken into account.

**Conclusion:**

The study provides estimates for *DSM-5* sexual dysfunction frequencies among Germans from a metropolitan area and points to the diagnostic value of age-related changes as well as the quality/avoidance of and discomfort during sex.

## Introduction

Links between sexual and general well-being are well documented^1^^,^^2^; accordingly, there is a definite need for tailoring adequate public health measures to strengthen sexual well-being. Knowledge on the size and burden of sexual problems and/or clinically relevant dysfunctions is, however, still limited and based on only a few studies with an adequate sample size and sampling strategy. Prevalence studies differ in how they operationalize the construct of *sexual dysfunction*. For example, not all studies account for the duration or psychological impact of sexual difficulties. It is possibly partially due to these inconsistencies that reported prevalence rates vary drastically between studies, even when influential factors like the country of study^3^ or the assessment tool^4^ are held constant. Furthermore, not factoring in the caused distress can lead to portrayals of a near epidemic spread of sexual dysfunctions. Numbers such as the ones from the much-cited study of Laumann et al,^5^ indicating that 43% of women and 31% of men in the United States experience sexual dysfunction, have been criticized for their contribution to an overmedicalization of sexuality, especially in the time of the rise of phosphodiesterase type 5 inhibitors such as Viagra.^6^^,^^7^ Still, many studies use the term *dysfunction* even when diagnostic criteria are not strictly assessed and subthreshold symptom manifestations included.

The two main diagnostic systems used to categorize sexual dysfunctions are the *International Classification of Diseases* (*ICD*) and the *Diagnostic and Statistical Manual of Mental Disorders* (*DSM*). Whereas the *ICD* seeks to classify various kinds of illnesses and health-related conditions and its 11th revision (*ICD-11*)^8^ groups sexual disorders of different etiology in a separate chapter (“conditions related to sexual health”), the *DSM* lists only mental disorders, thereby implying etiologic assumptions about the diagnosed conditions and excluding sexual dysfunctions attributed to somatic, cultural, or relationship factors. According to their comparison of sexual dysfunction diagnostics in the two systems, Schwesig et al^9^ emphasize that these conceptual differences hinder international communication and research cooperation. Moreover, the choice of diagnostic system affects research outcomes—for example, through creating more homogeneous research samples and thus reducing the generalizability to the total group of affected individuals when applying *DSM* criteria.^9^ At the same time, the clear benefit from using *DSM* criteria is that specific needs can be determined for individuals with sexual dysfunctions with no clear somatic, cultural, or relationship-based cause. This is of particular interest in older populations in which the incidence of chronic somatic complaints and associated sexual problems continuously rises, which might overshadow sexual dysfunctions arising from psychological factors.

The fifth edition of the *DSM* (*DSM-5*) includes female sexual arousal/interest disorder, female orgasmic disorder, genito-pelvic pain/penetration disorder, male hypoactive sexual desire disorder, erectile disorder, premature (early) ejaculation, and delayed ejaculation.^10^ It introduced important changes to the conceptualization of several categories and the omission of others as compared with the fourth edition of the *DSM* (text revision; *DSM-IV-TR*),^11^ and it added clear-cut morbidity criteria to focus sexual dysfunction diagnoses on clinically relevant complaints. For each diagnosis, symptoms must (A) be experienced 75% to 100% of the time, (B) last for at least 6 months, and (C) have caused significant distress. So far, these criteria have been used in only one study to determine the prevalence of sexual dysfunction, drawing on data from Britain’s third National Survey of Sexual Attitudes and Lifestyles (Natsal-3).^12^ When the rates of reported sexual difficulties (“experienced for 3 months or more in the last year”) were adjusted for the *DSM-5* morbidity criteria, they dropped from 22.8% to 3.6% in sexually active women and from 38.2% to 4.2% in sexually active men, emphasizing the importance of differentiating occasional, transient, or—from the perspective of affected individuals—more or less unproblematic experiences of sexual difficulties on one hand and lasting severe distress on the other.

This study aims to investigate the frequency of sexual dysfunctions as defined by *DSM-5* diagnostic criteria in a large population-based sample from the Hamburg City Health Study (HCHS; www.hchs.hamburg) and the factors associated with a diagnosis. Specifically, it focuses on the age cohorts between 45 and 74 years, in which the incidence of chronic somatic complaints and associated sexual problems continuously rises, thereby drawing more attention to somatic etiologic considerations. As such, our study will give insight into how the frequency of sexual dysfunctions according to the *DSM-5*—which includes only dysfunctions of psychological origin—among this age group compares with known prevalence rates from the general population.

The study addresses the following questions:

- Which frequency of sexual dysfunctions according to *DSM-5* criteria can be found in the population under study?
- Do participants with and without sexual dysfunction differ with regard to age, sex, quality ratings of their sexual relationships, or avoidance of sexual encounters?
- Can the fulfillment of sexual dysfunction criteria be predicted by quality ratings of participants’ sexual relationships, avoidance of sexual encounters, or discomfort during sex?

## Methods

### Study population

The HCHS is a single-center prospective observational cohort study with the purpose of deepening the understanding of disease development and survivorship from age 45 by using data from biological samples, medical examinations, modern imaging techniques, and self-report questionnaires.^13^ Participants stem from a sample randomly drawn from the official inhabitant data of the second largest city in Germany, which was divided into 6 age and gender strata. The final sample size is aimed to include 45 000 participants aged 45 to 74 years from the general population. To date, the estimated response rate is 23% to 30%, as nonresponders have not yet been identified among the most recent participants. The study protocol was approved by the ethics committee of the Hamburg Chamber of Medical Practitioners (Landesärztekammer Hamburg, PV5131).

All participants gave written informed consent after a nurse explained the study rationale to them at their baseline appointments. They filled out self-report questionnaires before, during, and after the 7-hour baseline examination, which included the items used in this study (see Jagodzinski et al^13^ for more details on the baseline examination). Of the first 10 000 participants at the baseline of the HCHS, all who answered at least one item of the questionnaire on sexual dysfunctions were included in this cross-sectional study.

### Instrument

A screening instrument for sexual dysfunctions was developed (Brief Questionnaire on Sexuality) that rigorously translates *DSM-5* criteria into questionnaire items.^14^ It consists of 6 sections covering the *DSM-5* A criteria of sexual dysfunctions with high face validity. To allow for a screening for female sexual interest/arousal disorder, a section on difficulties with lubrication in women was added. The frequency of sexual difficulties in the past 12 months is indicated on a 5-point Likert scale (not at all, rarely, sometimes, frequently [75%], always/nearly always). For difficulties that have been experiences rarely or more often, each of the following is registered by one dichotomous item: the distress caused by difficulties (C criterion); their duration (B criterion); and whether they are exclusively attributed to physical, psychological (ie, other mental health problems), and relationship problems and other distressing circumstances (D criterion). The questionnaire is a revised and *DSM-5*–adapted version of a previous instrument that showed favorable convergent validity with a structured clinical interview for sexual dysfunctions^15^ and was sensitive to changes over the course of psychotherapy.^16^ In the following, the term *sexual dysfunction* is used to refer to all queried sexual experiences that, in accordance with the *DSM-5* guidelines, occurred at least frequently (75%); lasted over at least 6 months in the last year; caused severe or very severe distress; and were not exclusively attributed to physical, psychological, or relationship problems/other distressing circumstances. *Sexual difficulties* describe all queried sexual experiences without taking those criteria into account.

Additionally, the questionnaire included one item on avoidance of sexual encounters, one on the quality of sexual relationships, and one on discomfort during sex.

### Statistical analysis

Data of participants with and without sexual dysfunction were compared with a Fisher test for categorical variables and a Mann-Whitney *U* test for continuous variables. To test the association of avoidance of sex and sexual relationship quality with the presence of sexual dysfunction (dependent variable), binary logistic regression models based on maximum likelihood estimation were calculated, adjusting for age and gender. Differences reaching *P* ≤ .05 were considered statistically significant. All statistical analyses were performed with R (version 4.0.3).

## Results

In total, we included 7786 participants in our study. Sample characteristics are displayed in Table 1.

**Table 1:** Sample characteristics (N = 7786).

	**No. (%)** ^a^
Age, y, median (IQR)	62.0 (55.0-69.0)
Women among participants	3980 (51.1)
Civil status	
Single	987 (12.7)
Married, living with spouse	4909 (63.0)
Married, living separated from spouse	187 (2.4)
Divorced	986 (12.7)
Widowed	454 (5.8)
I don’t know	36 (0.5)
I don’t want to answer	18 (0.2)
Missing	209 (2.7)
Education	
Currently in school	81 (1.0)
No degree	96 (1.2)
9 y of school	1480 (19.0)
8 or 9 y of school (in former GDR)	22 (0.3)
10 y of school	1882 (24.2)
10 y of school (in former GDR)	170 (2.2)
12 or 13 y of school	3610 (46.2)
I don’t know	62 (0.8)
I don’t want to answer	9 (0.1)
Missing	383 (4.9)
Occupation	
Full-time employed	2690 (34.5)
Part-time employed	997 (12.8)
Partial retirement	94 (1.2)
Employed below reporting threshold	266 (3.4)
Additional employment to unemployment benefits	6 (0.1)
Casually or irregularly employed	83 (1.1)
Apprenticeship	1 (<0.1)
Retraining	4 (0.1)
Parental leave or other leave	3 (<0.1)
Currently not employed^b^	2930 (37.6)
I don’t know	109 (1.4)
I don’t want to answer	28 (0.4)
Missing	575 (7.4)

Abbreviation: GDR, German Democratic Republic (East Germany).

^a^Percentages are rounded.

^b^Including student, unemployed, early retirement, retirement.

Frequencies for all sexual difficulties and dysfunctions according to *DSM-5* criteria are presented in Table 2. The overall frequency of sexual dysfunction was 9.3% (95% CI, 8.3%-10.4%) in women and 6.2% (95% CI, 5.4%-7.1%) in men. The most common sexual difficulties and dysfunctions in women concerned sexual interest and arousal (difficulty in the last 12 months, 42.5% [95% CI, 40.8%-44.1%]; sexual dysfunction, 6.8% [95% CI, 5.9%-7.7%]). For men, difficulties with erection were most prevalent (difficulty in the last 12 months, 15.6% [95% CI, 14.4%-16.9%]; sexual dysfunction, 3.5% [95% CI, 2.9%-4.1%]).

**Table 2:** Sexual difficulties and related diagnostic criteria in those reporting at least one sexual difficulty in the Hamburg City Health Study.

			**Of those who reported the difficulty occurring most of the time/always in the past 12 mo** ^**a**^									
	**12-mo prevalence** ^**b**^		**Severe distress**		**For at least 6 mo**		**Only with mental or physical health problem**		**Only with partner problem or distressing life situation**		**Fulfill *DSM-5* criteria for sexual dysfunction** ^**a**^	
	**No.**	**%** **(95% CI)**	**No.**	**%** **(95% CI)**	**No.**	**%** **(95% CI)**	**No.**	**%** **(95% CI)**	**No.**	**%** **(95% CI)**	**No.**	**%** **(95% CI)**
**Women**												
Lacked interest in having sex and/or arousal	1453	42.5 (40.8-44.1)	377	26.9 (24.6-29.3)	1285	92.8 (91.4-94.1)	256	18.6 (16.6-20.8)	429	31.3 (28.9-33.9)	221	6.8 (5.9-7.7)
Difficulty in reaching orgasm	467	14.5 (13.3-15.8)	120	29.1 (24.7-33.7)	356	87.5 (83.9-90.5)	81	19.8 (16-23.9)	115	28.3 (24-33)	51	1.6 (1.2-2.1)
Felt physical pain associated with intercourse	189	5.7 (4.9-6.5)	158	86.3 (80.5-91)	183	98.4 (95.4-99.7)	62	33.9 (27.1-41.2)	56	30.4 (23.9-37.6)	79	2.4 (1.9-3)
Experienced ≥1 of these	1630	49.4 (47.7-51.1)	478	30.8 (28.5-33.1)	1417	92.2 (90.7-93.5)	304	19.9 (17.9-22)	482	31.6 (29.3-34.1)	280	9.3 (8.3-10.4)
**Men**												
Lacked interest in having sex	466	13 (11.9-14.1)	91	20.7 (17-24.8)	394	90 (86.7-92.6)	135	31.1 (26.8-35.7)	144	33.3 (28.8-37.9)	26	0.7 (0.5-1.1)
Difficulty getting or keeping an erection	551	15.6 (14.4-16.9)	302	57.4 (53.1-61.7)	502	94.9 (92.7-96.6)	219	41.7 (37.5-46.1)	99	18.9 (15.7-22.6)	122	3.5 (2.9-4.1)
Difficulty in reaching ejaculation or orgasm	255	7.4 (6.5-8.3)	121	50 (43.5-56.5)	234	95.5 (92.1-97.7)	113	46.7 (40.3-53.2)	57	23.5 (18.3-29.3)	40	1.2 (0.8-1.6)
Premature ejaculation	293	8.5 (7.6-9.5)	140	49.5 (43.5-55.5)	264	93.6 (90.1-96.2)	53	18.9 (14.5-23.9)	51	18 (13.7-23)	81	2.4 (1.9-2.9)
Experienced ≥1 of these	1003	29.1 (27.6-30.7)	450	46.6 (43.4-49.8)	904	93.6 (91.8-95)	320	33.3 (30.3-36.3)	250	26.1 (23.3-29)	206	6.2 (5.4-7.1)

Abbreviation: *DSM-5*, *Diagnostic and Statistical Manual of Mental Disorders* (fifth edition).

^a^Percentages indicate relative frequencies among participants who answered the respective items; therefore, the n of the reference group may vary.

^b^Percentages are rounded, with the reference group being the number of participants who answered the item for each difficulty. Women: lacked interest in having sex, n = 3578; difficulty with lubrication, n = 3351; difficulty in reaching orgasm, n = 3211; felt physical pain associated with intercourse, n = 3330. Men: lacked interest in having sex, n = 3595; difficulty getting or keeping an erection, n = 3528; difficulty in reaching ejaculation or orgasm, n = 3467; premature ejaculation, n = 3431.

**Figure 1:**
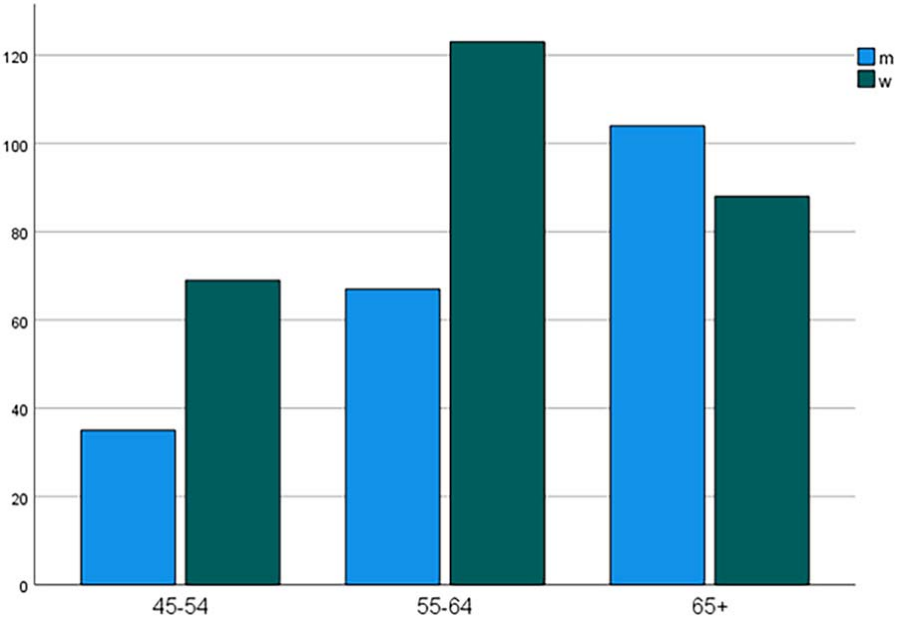
Distribution of women and men fulfilling *DSM-5* criteria for sexual dysfunction among age groups (n = 486). *DSM-5*, *Diagnostic and Statistical Manual of Mental Disorders* (fifth edition).

Whereas the number of women fulfilling diagnostic criteria for sexual dysfunction in comparison with men was twice as high in 45- to 54-year-olds and nearly twice as high in 55- to 64-year-olds, the share of men with a sexual dysfunction diagnosis visibly increased in ≥65-year-olds and surpassed the number of women with a sexual dysfunction diagnosis (Figure 1).

When compared with participants not fulfilling sexual dysfunction *DSM-5* criteria, those fulfilling these criteria were significantly older (*P* = .003), more often women (*P* = .012), rated the quality of their sexual encounters less often as very good/good and more often as insufficient (*P* < .001), and avoided sex more often (*P* < .001) (Table 3).

**Table 3:** Comparison of women and men with and without sexual dysfunction.

	**Sexual dysfunction, No. (%)**			
	**No (n = 5846)**	**Yes (n = 486)**	***U***	***P* value** ^**a**^
Age, y, median (IQR)	61.0 (54.0-68.0)	62.0 (55.0-69.0)	1 338 043	.033
Women	2725 (46.6)	280 (57.6)	—	<.001
Sexual quality^b^			—	<.001
1	511 (12.5)	13 (4.1)		
2	1548 (37.9)	80 (25.2)		
3	1255 (30.7)	116 (36.5)		
4	418 (10.2)	51 (16.0)		
5	233 (5.7)	37 (11.6)		
6	123 (3.0)	21 (6.6)		
Avoided sex^c^: yes	658 (14.7)	179 (46.6)	—	<.001

^a^Based on a Fisher test for categorical variables and Mann-Whitney *U* test for continuous variables.

^b^“Assume that there were grades for the sexual quality of a relationship, which grade from 1 to 6 (1 = very good, 6 = insufficient) would you give?” Responses: I didn’t have a sexual relationship in the past year, grades 1 to 6, I don’t know, I don’t want to answer.

^c^“During the past 12 months, have you avoided sex because of (one of) the mentioned problems?” Responses: no, yes, I don’t know, I don’t want to answer.

A sexual dysfunction diagnosis (weighted for age and gender) was significantly associated with the following: the avoidance of sex (χ^2^[3] = 207.8, *P* < .001), participants’ ratings of the quality of their sexual relationship(s) (reference: 2 = good; χ^2^[7] = 104.4, *P* < .001), and the experience of discomfort during sex (reference: not at all uncomfortable; χ^2^[6] = 186.7, *P* < .001). Participants who indicated avoiding sex were nearly 5 times more likely to fulfill criteria for sexual dysfunction (odds ratio, 4.936; 95% CI, 3.971-6.136). Those who rated the quality of their sexual encounters as insufficient (rating 6) were >3 times more likely to fulfill criteria for sexual dysfunction than participants who rated the quality of sex as good (rating 2; odds ratio, 3.353; 95% CI, 1.997-5.629). Finally, participants who indicated experiencing discomfort during sex always/nearly always were >7 times more likely to meet criteria for sexual dysfunction than those who did not experience any discomfort (odds ratio, 7.266; 95% CI, 3.769-14.007; Table 4).

**Table 4:** Logistic regressions.^a^

	***B***	***P* value**	**Odds ratio**	**95% CI**
Avoidance of sexual encounters (n = 4859)		<.001		
Avoidance: yes	1.597	<.001	4.936	3.971-6.136
Age	0.137	.145	1.146	0.954-1.377
Sex: male	−0.342	.002	0.710	0.570-0.885
Quality rating of sexual relationships (n = 4406)		<.001		
Grade: 1	−0.718	.018	0.488	0.269-0.885
Grade: 3	0.569	<.001	1.767	1.315-2.374
Grade: 4	0.831	<.001	2.296	1.587-3.321
Grade: 5	1.146	<.001	3.146	2.075-4.768
Grade: 6	1.210	<.001	3.353	1.997-5.629
Age	0.321	.001	1.378	1.131-1.678
Sex: male	−0.608	<.001	0.545	0.431-0.688
Discomfort during sex (n = 5548)^b^		<.001		
Rarely	0.641	<.001	1.899	1.473-2.448
Sometimes	1.561	<.001	4.761	3.646-6.218
Frequently (75%)	1.838	<.001	6.282	3.991-9.890
Always/nearly always	1.983	<.001	7.266	3.769-14.007
Age	0.130	.131	1.139	0.962-1.349
Sex: male	−0.398	.720	0.961	0.773-1.194

^a^Dependent variable: presence of sexual dysfunction according to DSM-5 criteria.

^b^“Have you experienced sex as uncomfortable?” Responses: not at all, rarely, sometimes, frequently (75%), always/nearly always.

## Discussion

Our results reveal a number of similarities to, but also differences from, Britain’s Natsal-3 (based on *DSM-5* guidelines)^12^ and Germany’s first representative national survey on sexual health (Gesundheit und Sexualität in Deutschland [GeSiD]; based on *ICD-11* guidelines),^17^ thus pointing to the significance of the examined target population and the assessment of *sexual dysfunction* criteria. Furthermore, our results confirm the association of the quality and avoidance of sex and discomfort during sex with a sexual dysfunction diagnosis.

The differences between Natsal-3 and HCHS results (e.g., 15% vs 13% for lack of sexual interest in men and 14.9% vs 8.5% for premature ejaculation) are possibly attributable to the survey instrument, with Natsal-3 based on a detailed validated questionnaire and HCHS based on a short screening instrument (the latter due to the number of medical topics that participants were asked about). Still, it is conceivable that the difference in rates of *dysfunction* stems from a difference between the study populations in experiencing and interpreting sexual difficulties, in openness to report distress, due to the influence of cultural contexts on sexual expression and experiences, or in the way that survey questions are perceived and reported.

Lower sexual dysfunction frequencies among HCHS participants for overall sexual dysfunction and most individual dysfunctions among women and men as compared with the GeSiD survey might be attributed to (1) the GeSiD sample being representative for Germany and the HCHS participants being recruited in a metropolitan area or (2) different criteria for “sexual dysfunction” being applied (GeSiD queried difficulties “over a period of several months”; HCHS referred to difficulties “over at least 6 months”). Additionally, *DSM* criteria refer only to mental conditions, hence leading to lower frequencies. Either way, these results underline the importance of considering such influential factors when interpreting frequency rates for sexual difficulties or dysfunctions.

Sexual interest/arousal disorders in women stood out as considerably more prevalent among HCHS participants than among those of Natsal-3 (6.8% vs 0.6%). This can be explained not only by the different age ranges of the samples but also by the difference in which symptoms were included between the studies: to approximate criteria for the *DSM-5*’s female sexual interest/arousal disorder, which requires the presence of 3 sexual symptoms out of a list, the Natsal-3 study estimated the prevalence of lack of sexual interest and arousal, whereas in this study we report the frequency of lacking interest and/or arousal (in our sample, the frequency of lacking sexual interest fulfilling all morbidity criteria was 0.7%, which matches the Natsal-3 data). Surveys including all listed symptoms, as well as studies assessing sexual dysfunctions by means of questionnaire data and structured clinical interviews, need to be conducted to determine the exact prevalence of female sexual interest/arousal disorder as defined by the *DSM-5*.

The finding from the GeSiD survey that a large share of women who experience pain in association with vaginal intercourse feel strongly impaired by it was confirmed by our data, emphasizing the necessity of devoting further research efforts to the diagnostics and treatment of women’s sexual pain.

There were obvious differences between women and men in the distribution of sexual dysfunctions across age groups. These results may be routed in differences in age-related bodily changes, such as health conditions affecting men’s sexual response (eg, heart conditions, diabetes) or menopause in women. However, next to physical change, the cultural framing of aging and sexuality needs to be taken into account, as well as how this framing differs between women and men.

Quality ratings and avoidance of sex, as well as the experience of sex to be uncomfortable, were clearly correlated with the presence of sexual dysfunction as defined by the *DSM-5*. This again demonstrates how intimately sexual dysfunction is linked to sexual behavior, relationship quality, and well-being. The assessment of these factors, which can be implemented time-economically in different clinical settings, can promote the detection of sexual dysfunction in patients more generally.

Finally, a general question needing to be addressed is whether the *DSM-5* D criterion—which excludes sexual symptoms exclusively based on physical, other psychological, or relationship problems—can adequately be assessed via self-report. In contrast to Natsal-3, in which prevalence figures do not take the D criterion into account because of the difficulty of verifying it through cross-sectional self-report data,^12^ this study employed a screening tool that aims to approximate it for every present symptom. Validation studies with clinically diagnosed samples are required to more closely examine the screening tool’s sensitivity and specificity regarding the *DSM-5* diagnoses.

Further limitations to the interpretation and generalizability of the results presented here include the restriction to self-report questionnaire data—since sexual dysfunctions are best diagnosed via structured clinical interviews, self-report questionnaire data can at most be interpreted as an approximation of prevalence. In addition, the generalizability was limited by the focus on participants living in a metropolitan area—resulting in, for example, a higher grade of education: 46.2% of participants graduated from school after 12 or 13 years vs 33% in the general German population (cf Federal Ministry of Education and Research^18^). Furthermore, the analysis does not account for whether participants did not experience sexual difficulties because they were not sexually active in the queried period, nor does it factor in the potential bias from participant exclusion or nonresponse during data collection. Still, the large study sample grants the data robustness to give valuable insight on the frequency of sexual difficulties and dysfunctions as defined by the *DSM-5* criteria.

## Conclusion

In line with the results from Natsal-3, our data confirm the importance of applying diagnostic criteria, such as recurrence/persistence of symptoms or distress from them, when reporting the frequency of sexual *dysfunction*. *DSM-5* criteria have been criticized for complicating access to treatment for individuals experiencing sexual difficulties through the added requirements for duration and frequency,^19-21^ and it remains questionable to which degree examiners and patients can reliably distinguish psychological from somatic developmental factors. Nevertheless, an emphasis on frequent and lasting sexual difficulties represents a protection against a superficial medicalization of sexual expression in its variety and variability. Beyond this, the comparison of our data with those from existing prevalence studies corroborates the necessity of detailed transparent reporting of sample selection and measurement criteria in research on the prevalence of sexual difficulties and dysfunction. For clinical practice, our data suggest that sexual dysfunction caused by other than physical health or relationship factors is important to consider among 45- to 74-years-olds and that avoidance of, perceived quality of, and discomfort during sex serve as valuable diagnostic indicators for the presence of sexual dysfunction. Moreover, the study demonstrates the applicability of a short screener for sexual dysfunctions according to *DSM-5* criteria, which can be implemented in clinical practice as a starting point for sexual history taking.

## Acknowledgments

The authors acknowledge the participants of the Hamburg City Health Study, the staff at the Epidemiological Study Centre, cooperation partners, patrons and the Deanery from the University Medical Centre Hamburg.

## Funding

The participating institutes and departments from the University Medical Center Hamburg-Eppendorf contribute with individual and scaled budgets to the overall funding. The study is also supported by the Innovative medicine initiative (IMI) under Grant No. 116074, by the Fondation Leducq under Grant Number 16 CVD 03, by the euCanSHare Grant Agreement No. 825903-euCanSHare H2020 and the DFG under project Grant TH1106/5-1; AA93/2-1. The licence for the Food Frequency and Physical activity is provided by the DIFE. Technical equipment is provided by SIEMENS according to a contract for 12 years as well as by the Schiller AG on a loan basis for 6 years and by Topcon on a loan basis from 2017 until 2022. The Hamburg City Health Study is additionally supported by an unrestricted Grant (2017–2022) by Bayer. Project related analyses are supported by Amgen, Astra Zeneca, BASF, Deutsche Gesetzliche Unfallversicherung (DGUV), DKFZ, DZHK, Novartis, Seefried Stiftung and Unilever. The study is further supported by donations from the “Förderverein zur Förderung der HCHS e.V.”, TEPE (2014) and Boston Scientific (2016). A current list of the supporter is online available on www.uke.de/hchs.

*Conflicts of interest:* Peer Briken was an advisor to the WHO with regard to the classification of sexual disorders in ICD-11. The remaining authors declare that there is conflict of interest.
